# Injury of the Face

**Published:** 1875-04

**Authors:** J. S. Thurnan


### ARTICLE VII.

### *Injury of the Face.*

BY J. S. THURNAN, M. D.

I am prompted to report the following case, on account of its rarity in country practice. In Southwest Missouri physicians seldom meet with cases requiring immediate surgical appliances.

I was called in great haste, on the 5th of January last, to see Elois Acock, aged twenty four years. On my arrival I found him lying on the floor in a semi-conscious state, with copious hemorrhage flowing from the mouth and nose. I learned, upon inquiry, that he was passing near the heels of a horse, and slapped it with his hand, at which the animal became frightened, and kicked with all its force, striking patient on the right side of the superior maxillary bone and mouth. I found, upon examination, the upper jaw to be fractured, in a line commencing just behind the last molar tooth, and passing across the malar process to the lateral boundary of the anterior nares, and then down between the canine and incisor teeth. On manipulation of the left side of the upper jaw, I detected mobility and crepitation, but was unable to determine the line of fracture; apparently all the bones of the face were detached and the soft parts terribly contused.

The swelling soon closed his eyes; then he was a horrible looking object. There was a gash cut in the lower lip, I presume by the shoe of the horse, three-fourths of an inch in length. Reaction came on in three or four hours. The hemorrhage was soon controlled by the application of cold water. Several small spicula of detached bone were removed with dressing forceps. The fractured part was then adjusted by introducing my finger into his mouth and placing and holding the detached portion until a cork was placed between the first molars on each side, and the lower jaw brought firmly up, which held the cork in its place; a bandage was then applied, such as is used in fracture of the inferior maxillary. The gash in the lip had previously been united with sutures; the other bones were replaced as far as possible. Patient was then placed in bed, the mouth and nose ordered washed with solution of carbolic acid four times per day, and fluid nourishment. The first twenty-four hours patient was very restless, after which he became more quiet and rested well. I removed the bandage on the 14th day, and found union perfect. Some discharge from the nose. Continued carbolic acid solution to the nose. Four weeks after, I saw the patient at my office; there was no discharge from the nose and he said he was satisfied with his jaw, but should have been glad of a few more teeth.—*Medical and Surgical Reporter.*

---

